# Effect of Glutamine on the Growth Performance, Oxidative Stress, and Nrf2/p38 MAPK Expression in the Livers of Heat-Stressed Broilers

**DOI:** 10.3390/ani13040652

**Published:** 2023-02-13

**Authors:** Xi Bai, Kunping Wang, Rifat Ullah Khan, Cheng Zhang, Hong Hu

**Affiliations:** 1 College of Animal Science, Anhui Science and Technology University, Chuzhou 233100, China; 2 College of Veterinary Sciences, the University of Agriculture, Peshawar 25130, Pakistan; 3 College of Animal Science and Technology, Anhui Agricultural University, Hefei 230036, China

**Keywords:** broiler, glutamine, heat stress, oxidative stress, Nrf2, p38 MAPK, liver

## Abstract

**Simple Summary:**

Dietary glutamine (Gln) supplementation can significantly alleviate the negative effects on product performance and antioxidant capacity in chickens exposed to hot environments. However, the antioxidant effect of Gln on the liver in heat-stressed broilers and its mechanism of action are rarely reported. The aim of the present study was to investigate the effect of glutamine on the growth performance, oxidative stress, and Nrf2/p38 MAPK single pathway in the livers of broilers exposed to high temperature conditions. Results showed that high temperature conditions decreased growth performance and induced oxidative stress in broiler livers. Dietary Gln improved the growth performance, antioxidant enzyme, Nrf2, and p38 MAPK expression in the livers of heat-stressed broilers. In conclusion, this study suggested that Gln can improve the liver’s response to oxidative stress by increasing the Nrf2/p38 MAPK expression. Gln can be used as a feed additive for broiler production in high-temperature environments such as during the summer season.

**Abstract:**

The purpose of this work was to study the effects of glutamine (Gln) on the growth performance, oxidative stress, Nrf2, and p38 MAPK pathway in the livers of heat-stressed broilers. In total, 300 broilers were divided into five groups, including a normal temperature (NT, without dietary Gln) group and four cyclic high temperature groups (HT, GHT1, GHT2, and GHT3) fed with 0%, 0.5%, 1.0%, and 1.5% Gln, respectively. High temperature conditions increased (*p* < 0.05) liver malonaldehyde (MDA) concentration, but decreased (*p* < 0.05), body weight gain (BWG), feed intake (FI), liver superoxide dismutase (SOD), total antioxidant capacity (T-AOC), glutathione peroxidase (GSH-Px), glutathione S-transferase (GST), and glutathione (GSH) levels in broilers. Nrf2 and p38 MAPK protein and mRNA expression levels were lower (*p* < 0.05) in the NT group than that in the HT group. However, dietary 1.5% Gln decreased (*p* < 0.05) liver MDA concentration, but increased (*p* < 0.05) BWG, FI, liver SOD, T-AOC, GSH-Px, GST, and GSH levels in heat-stressed broilers. Nrf2 and p38 MAPK protein and mRNA expression levels were higher (*p* < 0.05) in the GHT3 group than that in the HT group. In summary, Gln improved oxidative damage through the activation of Nrf2 and p38 MAPK expression in the livers of heat-stressed broilers.

## 1. Introduction

Heat stress is the most important environmental stress factors in livestock and poultry production worldwide [1]. Heat stress is caused by the imbalance between heat production and heat dissipation, that is, when heat produced by the animal body is greater than the heat released to the environment. Heat stress causes great loss to livestock and poultry production. Heat stress leads to increased metabolism and decreased feed intake, and ultimately results in the decreased growth, production, and reproductive performance of livestock and poultry [2]. Heat stress can enhance the metabolism of livestock and poultry, cause excessive accumulation of reactive oxygen species, and cause an oxidation–antioxidant imbalance in the body, resulting in oxidative stress [3,4]. Many studies have shown that long-term heat stress reduces antioxidant capacity and causes tissue oxidative damage in livestock and poultry [3,4]. The damage of heat stress can be effectively reduced by feeding functional additives.

Glutamine (Gln) is a conditional essential amino acid with a wide range of biological functions [5]. Gln is important in promoting production performance, maintaining intestinal health, enhancing immunological responses, and preventing damage from oxidation reactions in livestock [6,7,8]. Furthermore, Gln can significantly improve the abnormal physiological functions resulting from cold temperature, heat, immunological, weaning, transportation, and oxidative stresses [9]. Previous studies have shown that Gln not only provides energy for the growth of intestinal mucosal cells, but also participates in the synthesis of glutathione (GSH) and the process of redox and oxidative free radical scavenging in the body [9,10]. Upon stressful conditions in an organism, the need for Gln increases, resulting in a decrease in both Gln levels and antioxidant capacity [9].

Broiler chickens have thick skin covered with feathers, more subcutaneous fat, fewer developed sweat glands, slower heat dissipation on the body surface, and often have difficulty tolerating higher temperatures [11,12]. The liver is an important metabolic organ in broiler chickens, and its main functions are deoxidation, detoxification, regulation of fat metabolism and electrolytes, and storage of glycogen [13]. Broiler liver cells synthesize various antioxidant enzymes at a relatively constant rate at room temperature. These antioxidant enzymes can breakdown peroxides into less toxic or harmless chemicals, which act as a barrier for the body. The heat-stress environment significantly increases oxidative damage to the bioactive macromolecules in broiler liver cells, reducing the biological activity of antioxidant molecules [14,15]. As a result, liver cells will be in a state of oxidative stress that would disrupt metabolic function and affect product performance. The organism may present with liver diseases and is at a greater risk of mortality.

Dietary Gln supplementation can significantly alleviate the negative effects on product performance and antioxidant capacity in chickens exposed to hot environments [16,17,18]. However, the antioxidant effect of Gln on the liver in heat-stressed broilers and its mechanism of action are rarely reported. Therefore, this experiment aimed to study the effect of Gln on oxidation performance, especially focusing on the mechanisms of nuclear factor erythroid 2–related 2/Kelch-like ECH-associated protein 1(Nrf2) and p38 mitogen-activated protein kinase (p38 MAPK) expression in the livers of broilers subjected to cyclic high temperature conditions.

## 2. Materials and Methods

### 2.1. Broilers and Sampling

The experiment was performed in accordance with the approval by the Animal Care and Use Committee of Anhui Science and Technology University. In total, 300 22-day-old Arbor Acres broilers (similar weight) from the Farm of Anhui Science and Technology University were allocated to five treatments (six cages and 10 broilers per treatment). These treatments included: NT group, normal temperature without dietary Gln; HT group, cyclic high temperature without dietary Gln; GHT1 group, cyclic high temperature and dietary 0.5% Gln; GHT2 group, cyclic high temperature and dietary 1.0% Gln; GHT3 group, cyclic high temperature and dietary 1.5% Gln. Birds in the NT group were housed in a normal environment at 24 °C per day. Birds in the HT, GHT1, GHT2, and GHT3 groups were housed in cyclic high-temperature environments at 34 °C for 8 h and 24 °C for 16 h. The duration of this experiment was 21 days. The basal diet (corn-soybean meal) designed according to NRC [19] is shown in Supplementary Table S1, as described by Hu et al. [20]. Eighteen birds from each group (3 birds per cage) were euthanized on day 42. Liver samples were collected and stored at −70 °C.

### 2.2. Detection of Growth Performance

The body weight gain (BWG) and feed in take were determined on days 28 and 42 of age and the feed-to-gain ratio (FGR) wascalculated as FI/BWG.

### 2.3. Detection of Antioxidant Status

Malonaldehyde (MDA), superoxide dismutase (SOD), catalase (CAT), and total antioxidant capacity (T-AOC) were detected using the following assay kits: MDA (TBA method), SOD, and T-AOC assay kits (Jiancheng company, Nanjing, China).

### 2.4. Detection of GSH-Related Enzymes

GSH-related enzymes were detected using the following assay kits: Glutathione S-transferase (GST), glutathione peroxidase (GSH-Px), and Glutathione (GSH) assay kits (Jiancheng company, Nanjing, China).

### 2.5. Detection of Nrf2 and p38 MAPK Concentrations

Nrf2 and p38 MAPK were detected using the Nrf2 and p38 MAPK (Elisa method) assay kits, respectively. These assay kits were produced at the Jiancheng company (Nanjing, China).

### 2.6. Expression of Nrf2 and p38 MAPK mRNA

Quantitative Real-Time PCR (qRT-PCR) was used to detect the expression of Nrf2 and p38 MAPK mRNA. Total RNA was extracted from the liver by a total RNA kit (Tiangen company, Beijing, China). cDNA was prepared using a cDNA synthesis kit (TaKaRa). The β-actin was used as a housekeeping gene. The following primers were used: β-actin (F: 5′- TGCTGTGTTCCCATCTATCG -3′; R: 5′- TTGGTGACAATACCGTGTTCA -3′), Nrf2 (F: 5′- TCGCAGAGCACAGATAC -3′; R: 5′- AGAAATGAAGACTGGGTC -3′), and p38 MAPK (F: 5′- AAGGTTGGCAAGCATGAGTT -3′; R: 5′- TTCTGGGCCTGCATATAACC -3′). The reaction (20 μL system) and program of qRT-PCR were performed as previously described by Hu et al. [20]. The mRNA expression level of Nrf2 and p38 MAPK mRNA was measured by the 2^−ΔΔCt^ method, which normalized to β-actin Ct.

### 2.7. Statistical Analysis

Data which included NT, HT, GHT1, GHT2, and GHT3 groups were calculated using one-way ANOVA in SPSS 18.0 software. The statistical difference was evaluated by Duncan’s test. The *p* < 0.05 was regard as statistically significant. The data was presented as mean ± standard error of the mean (SEM)

## 3. Results

### 3.1. Effects of Gln Supplement on Growth Performance of Heat-Stressed Broilers

**Figure 1:**
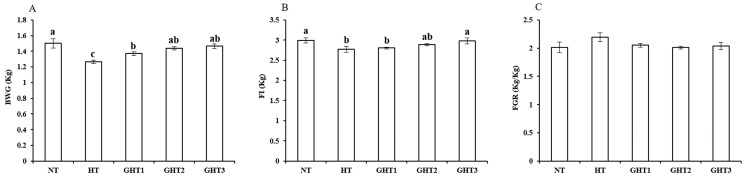
Effect of Gln on growth performance of broilers exposed to high temperature conditions. (**A**) BWG; (**B**) FI; (**C**) FGR. Different lowercase letters above each column indicate significant (*p* < 0.05) differences among the groups. NT group: Broilers were kept in a normal temperature environment and fed a basal diet. HT, GHT1, GHT2, and GHT3 groups: Broilers in these groups were kept in high temperature conditions and fed a basal diet supplemented with 0%, 0.5%, 1.0%, and 1.5% Gln.

Figure 1 shows the effects of Gln supplement on growth performance of broilers exposed to high temperature conditions. Cyclic high-temperature conditions decreased (*p* < 0.05) BWG and FI in broilers. The GHT3 group had higher (*p* < 0.05) BWG and FI than that in the HT group in broilers (Figure 1). However, there were no significant differences in the BWG and FI among the NT, GHT2 and GHT3 groups.

### 3.2. Effects of Gln Supplement on MDA on the Livers of Heat-Stressed Broilers

**Figure 2:**
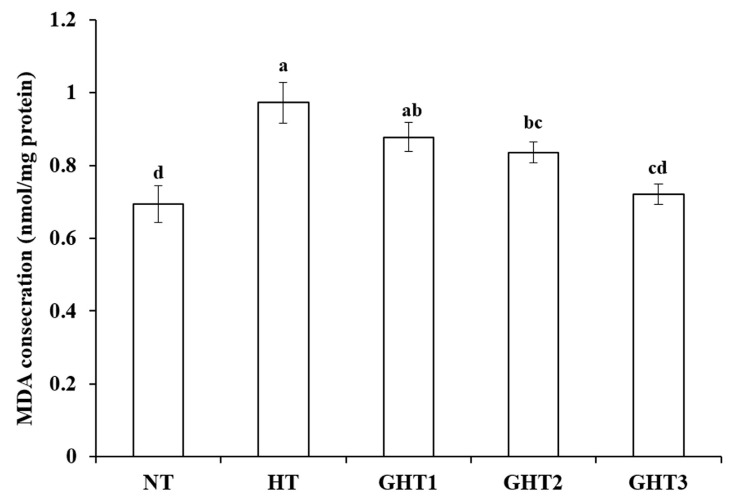
Effect of Gln on MDA level on the livers of broilers exposed to high temperature conditions. Different lowercase letters above each column indicate significant (*p* < 0.05) differences among the groups. NT group: Broilers were kept in a normal temperature environment and fed a basal diet. HT, GHT1, GHT2, and GHT3 groups: Broilers in these groups were kept in high temperature conditions and fed a basal diet supplemented with 0%, 0.5%, 1.0%, and 1.5% Gln.

Figure 2 shows the effects of Gln supplement on MDA on the livers of broilers exposed to high temperature conditions. Cyclic high-temperature conditions increased (*p* < 0.05) MDA concentration in broiler livers. The GHT2 and GHT3 groups had lower (*p* < 0.05) MDA levels than that in the HT group in broiler livers. However, there were no significant differences in the MDA between the NT and GHT3 groups.

### 3.3. Effects of Gln Supplement on SOD, CAT, and T-AOC on the Livers of Heat-Stressed Broilers

**Figure 3:**
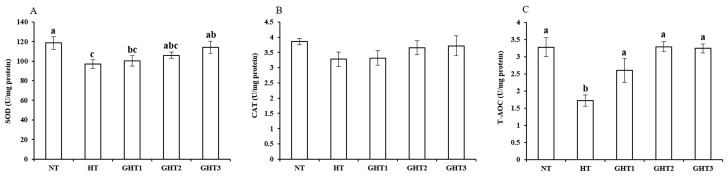
Effect of Gln on SOD (**A**), CAT (**B**), and T-AOC (**C**) levels on the livers of broilers exposed to high temperature conditions. Different lowercase letters above each column indicate significant (*p* < 0.05) differences among the groups. NT group: Broilers were kept in the normal temperature environment and fed a basal diet. HT, GHT1, GHT2, and GHT3 groups: Broilers in these groups were kept in high temperature conditions and fed a basal diet supplemented with 0%, 0.5%, 1.0%, and 1.5% Gln.

Figure 3 shows the effects of Gln supplement on SOD, CAT, and T-AOC on the livers of broilers exposed to high temperature conditions. Cyclic high-temperature conditions decreased (*p* < 0.05) the SOD and T-AOC levels in broiler livers. The SOD levels were higher (*p* < 0.05) in the GHT3 group than that in the HT group; the T-AOC levels were higher (*p* < 0.05) in the GHT1, GHT2, and GHT3 groups than that in the HT group in broiler livers. However, there were no significant differences in the SOD and T-AOC between the NT and GHT3 groups.

### 3.4. Effects of Gln Supplement on GSH-Related Enzymes on the Livers of Heat-Stressed Broilers

**Figure 4:**
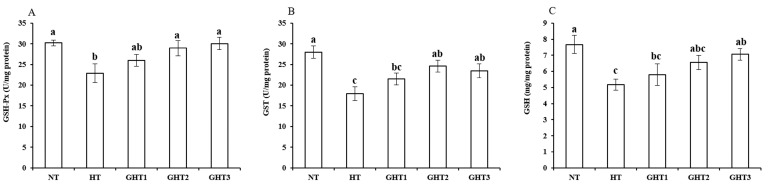
Effect of Gln on GSH-Px (**A**), GST (**B**), and GSH (**C**) levels on the livers of broilers exposed to high temperature conditions. Different lowercase letters above each column indicate significant (*p* < 0.05) differences among the groups. NT group: Broilers were kept in a normal temperature environment and fed a basal diet. HT, GHT1, GHT2, and GHT3 groups: Broilers in these groups were kept in high temperature conditions and fed a basal diet supplemented with 0%, 0.5%, 1.0%, and 1.5% Gln.

Figure 4 shows the effects of Gln supplement on GSH-related enzymes on the livers of broilers exposed to high temperature conditions. Cyclic high-temperature conditions decreased (*p* < 0.05) the GSH-Px, GST, and GSH concentrations in broiler livers. The GSH-Px and GST levels were higher (*p* < 0.05) in the GHT2 group than that in the HT group; the GSH-Px, GST, and GSH levels were higher (*p* < 0.05) in the GHT3 group than that in the HT group in broiler livers. However, there were no significant differences in the GSH-Px, GST, and GSH concentrations between the NT and GHT3 groups.

### 3.5. Effects of Gln Supplement on Nrf2 Protein and mRNA Expression on the Livers of Heat-Stressed Broilers

**Figure 5:**
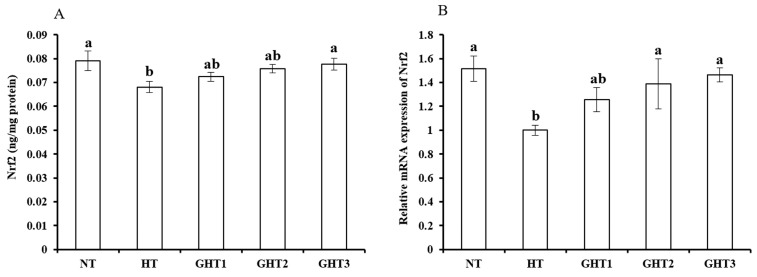
Effect of Gln on Nrf2 protein (**A**) and gene (**B**) expression in the liver of broiler exposed to high temperature conditions. Different lowercase letters above each column indicate significant (*p* < 0.05) differences among the groups. NT group: Broilers were kept in the normal temperature environment and fed a basal diet. HT, GHT1, GHT2, and GHT3 groups: Broilers in these groups were kept in high temperature conditions and fed a basal diet supplemented with 0%, 0.5%, 1.0%, and 1.5% Gln.

Figure 5 shows the effects of Gln supplement on the Nrf2 protein and mRNA expression on the livers of broilers exposed to high temperature conditions. Cyclic high-temperature conditions decreased (*p* < 0.05) the protein and mRNA levels of Nrf2 in the broilers livers. The protein and mRNA levels of Nrf2 were higher (*p* < 0.05) in the GHT3 group than that in the HT group (Figure 5). However, there were no significant differences in the Nrf2 protein and mRNA expression levels between the NT and GHT3 groups.

### 3.6. Effects of Gln Supplement on p38 MAPK Protein and mRNA Expression on the Livers of Heat-Stressed Broilers

**Figure 6:**
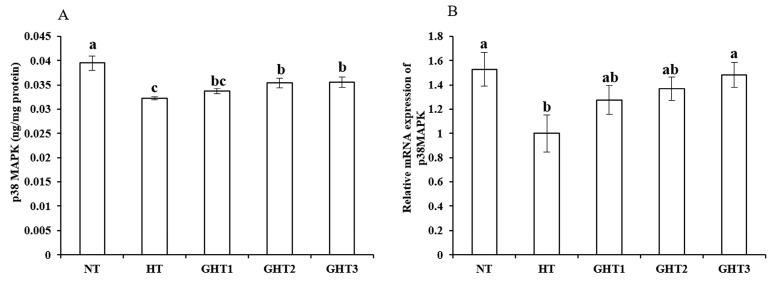
Effect of Gln on p38 MAPK protein (**A**) and gene (**B**) expression in the liver of broilers exposed to high temperature conditions. Different lowercase letters above each column indicate significant (*p* < 0.05) differences among the groups. NT group: Broilers were kept in the normal temperature environment and fed a basal diet. HT, GHT1, GHT2, and GHT3 groups: Broilers in these groups were kept in high temperature conditions and fed a basal diet supplemented with 0%, 0.5%, 1.0%, and 1.5% Gln.

Figure 6 shows the effects of Gln supplement on p38 MAPK protein and mRNA expression on the livers of broilers exposed to high temperature conditions. Cyclic high-temperature conditions decreased (*p* < 0.05) the protein and mRNA levels of p38 MAPK in the broilers livers. The protein and mRNA levels of p38 MAPK were higher (*p* < 0.05) in the GHT3 group than that in HT group. However, there were no significant differences in the p38 MAPK mRNA expression levels between the NT and GHT3 groups.

## 4. Discussion

When the broilers suffered from heat stress, the feed intake was reduced, which decreased the body weight gain, and then increased the catabolism of proteins, fats, and carbohydrates [21,22]. This biochemical response is intended to increase energy production to resist stress. The present study showed that growth performance was negatively affected by high temperature. Hu et al. also suggested that heat stress markedly decreased body weight gain, feed intake, and feed efficiency of broilers exposed to hot environments [22]. Gln, an important amino acid, is found abundantly in animals, including broiler chickens. It has unique functions in various organs. Gln not only provides energy for certain cells and tissues, but also provides precursors for various amino acid synthesis and maintains the balance of cell oxidation systems [20]. As a neutral and multifunctional essential amino acid, Gln is particularly prominent in the anti-stress response [23]. With the loss of nutrients, the Gln content in broiler tissues decreases significantly to the extent of being unable to fulfill the body’s needs during heat stress [20]. Upon exogenous Gln supplementation, the growth performance in broilers is significantly improved [24]. Similarity, the performance of heat-stressed broilers was reversed by dietary Gln in this study.

Heat stress reduces the intake of feed and the absorption of nutrients in broilers [25,26]. As a result, the body’s metabolic levels were affected, producing excessive free radicals, leading to disorders in antioxidant function [27]. When an animal is subjected to heat stress, the body temperature rises, affecting the metabolic enzyme activity and increasing the metabolic rate [26]. The high metabolic rate dramatically increases oxygen free radical production. Several studies have shown that the liver is susceptible to oxidative stress [28,29]. In the present study, heat stress increased the MDA content and significantly decreased antioxidant enzyme activities, suggesting that heat stress leads to liver oxidative damage in broilers. MDA, a final product of lipid oxidation, can aggravate the damage to the cell membrane, and affect the activities of mitochondrial respiratory chain complexes and key mitochondrial enzymes [30]. Similarly, Tang et al. showed that heat stress could increase MDA levels and cause oxidative stress in the broiler livers [30].

Redox reactions are among the most important physiological activities of the body. Generally, cells have a dynamic balance between the oxidation and antioxidant systems. This balance can remove superoxide anions to protect tissues and cells from damage. However, an imbalance in this system leads to abnormal functions in all aspects of the body [31]. Gln is a precursor for the synthesis of reduced glutathione [32]. The decrease in Gln due to the stress response is the rate-limiting factor for the synthesizing reduced glutathione [24]. Therefore, Gln can affect the antioxidant activities in vivo via GSH. GSH-Px activity reflects the degree of oxidative damage. CAT is the main enzyme involved in cellular H_2_O_2_ removal. SOD can prevent cellular oxidative damage caused by oxygen free radicals, effectively repairing damaged cells. This study found that Gln supplementation in the diet of heat-stressed broilers promoted antioxidant enzyme activities, but decreased the concentration of MDA in the liver, indicating that Gln could alleviate oxidative damage caused by heat stress.

Heat stress can lead to increased free radicals and oxidative damage in the liver [28,33]. Antioxidative responsive elements (ARE) can prevent peroxidation of lipid and reduce oxidative damage. Nrf2 is an important activator of ARE in the cellular oxidative stress response [34]. Long-term stress leads to the rapid exhaustion of Nrf2 stored in the body and decreases its expression levels. Zhang et al. suggested that high temperature conditions significantly decreased Nrf2 expression levels and antioxidant enzyme activities in broiler muscles [33]. Similar results were obtained in this study, whereby cyclic high-temperature environment markedly decreased the gene expression and concentration of Nrf2 in broiler liver cells. These experiments suggested that a high temperature environment inhibited the Nrf2 expression in broiler livers.

Nrf2 is expressed in many tissues and plays a key function in anti-oxidative stress [35,36]. Nrf2-Keap1 disassociates to release Nrf2 under stressful conditions, increasing Nrf2 expression levels, and enhancing the cell’s antioxidant ability. Gln can increase the synthesis of GSH in the mitochondria of hepatocytes and maintain the concentration of GSH in the liver tissue and plasma, thereby improving its antioxidant ability [37]. The present work indicated that Gln reduced oxidative stress through the Nrf2 signal pathway in broiler livers under high temperature conditions. Hu et al. showed that Gln increases Nrf2 levels in the leg muscles of broilers under heat stress, thereby improving oxidative damage [38].

As a member of the MAPK family, p38 MAPK can be activated under the stimulation of the external environment, thus regulating the expression of a series genes, and participating in the regulation process of heat stress [39]. The main function of p38 APK is to transfer cytoplasmic signals into the nucleus through phosphorylation of p38 protein to activate downstream gene transcription and trigger cell biological reactions. In this study, long-term thermal stress significantly reduced the expression level of p38 MAPK. Similar results were found in the study by Hu et al. [40]. However, dietary Gln can activate the p38 MAPK signaling pathway. Many studies revealed that Nrf2 is positively correlated with MAPK [41]. Therefore, the results of this study suggest that Gln improved the oxidative damage of the liver in broilers under heat stress, which may be related to the activation of Nrf2 and p38 MAPK.

## 5. Conclusions

For broilers, high-temperature conditions induced performance and oxidative damage in their livers. High-temperature conditions markedly decreased Nrf2 and p38 MAPK expression in the livers of broilers. However, this study shows that dietary 1.5% Gln can improve the liver’s response to oxidative stress by activating Nrf2 and p38 MAPK expression in the heat-stressed broiler. Gln can be used as a feed additive for broiler production in high-temperature environments such as during the summer season.

## Data Availability

The data presented in this study are available on request from the corresponding author. The data are not publicly available due to policy.

## Supplementary Materials

The following supporting information can be downloaded at https://www.mdpi.com/article/10.3390/ani13040652/s1, Table S1: Composition of the basal diets.
