# Supplementary material for: Effect of Glutamine on the Growth Performance, Oxidative Stress, and Nrf2/p38 MAPK Expression in the Livers of Heat-Stressed Broilers
Source: Animals (Basel). 2023 Feb 13;13(4):652. doi: 10.3390/ani13040652 (PMC9951748; doi:10.3390/ani13040652)
Supplement: Supplementary file 1 [file animals-13-00652-s001.zip › animals-2184042-supplementary.pdf]

Table S1 The basal diets in the present study

Ingredients (%)		Chemical composition	
Maize	58.5	ME (MJ/kg)	12.73
Starch	1.0	Crude Protein (%)	20.30
Soybean meal	32.0	Lysine (%)	1.08
Fish meal	2.0	Methionine+cysteine (%)	0.76
Soybean oil	3.5	Ca (%)	0.89
$CaHPO_4·2H_2O$	1.5	Available P (%)	0.42
Limestone	0.9		
Salt	0.3		
DL-Met	0.1		
Premix[1]	0.2		
Total	100		

[1] Provided per kilogram of diet: Cu (from $CuSO_4$): 8 mg; Zn (from $ZnSO_4$), 40 mg; Fe [from $Fe_2(SO_4)_3$]: 80 mg; I (from IK): 0.35 mg; Se (from Na_2SeO_3): 0.15 mg; vitamin B6: 3.0 mg; vitamin B12: 0.014 mg; vitamin E: 20 IU; vitamin K: 2.0 mg; vitamin A: 10,000 IU; cholecalciferol: 2600 IU; thiamin: 1.6 mg; riboflavin, 6.0 mg; calcium pantothenate: 20 mg; niacin: 30 mg; folic acid: 0.8 mg; biotin 0.12 mg; choline (as choline chloride), 500 mg.
